# The miR-24-3p/p130Cas: a novel axis regulating the migration and invasion of cancer cells

**DOI:** 10.1038/srep44847

**Published:** 2017-03-24

**Authors:** Hoin Kang, Jun Gi Rho, Chongtae Kim, Hyosun Tak, Heejin Lee, Eunbyul Ji, Sojin Ahn, A-Ri Shin, Hyun-Il Cho, Yun Hyun Huh, Woo Keun Song, Wook Kim, Eun Kyung Lee

**Affiliations:** 1 Department of Biochemistry, College of Medicine, The Catholic University of Korea, Seoul, South Korea; 2 Department of Molecular Science and Technology, Ajou University, Suwon, South Korea; 3 Catholic Cancer Research Institute, College of Medicine, The Catholic University of Korea, South Korea; 4 Department of Life Science, Bio Imaging and Cell Dynamics Research Center, Gwangju Institute of Science and Technology, Gwangju, South Korea; 5 Cancer Evolution Research Center, The Catholic University of Korea, Seoul, South Korea

## Abstract

MicroRNAs (miRNAs) are small non-coding RNAs that negatively regulate gene expression by suppressing translation or facilitating mRNA decay. Differential expression of miRNAs is involved in the pathogenesis of several diseases including cancer. Here, we investigated the role of-miR-24-3p as a downregulated miRNA in metastatic cancer. miR-24-3p was decreased in metastatic cancer and lower expression of miR-24-3p was related to poor survival of cancer patients. Consistently, ectopic expression of miR-24-3p suppressed the cell migration, invasion, and proliferation of MCF7, Hep3B, B16F10, SK-Hep1, and PC-3 cells by directly targeting p130Cas. Stable expression of p130Cas restored miR-24-3p-mediated inhibition of cell migration and invasion. These results suggest that miR-24-3p functions as a tumor suppressor and the miR-24-3p/p130Cas axis is a novel factor of cancer progression by regulating cell migration and invasion.

Cell migration is an integrated process that plays important roles in both physiological and pathological conditions^1^. Tight regulation of adhesion turnover is critical for the migration, invasion and metastasis of cancer cells^2^,^3^. Since metastasis is a leading cause of cancer-related deaths, several efforts have been made to overcome cancer metastasis. However, metastasis remains a common feature of malignancy and is often associated with poor prognosis^4^. In addition, the alteration of gene expression and cellular signaling responsible for metastasis is not fully elucidated.

microRNAs (miRNAs) are small non-coding RNAs that function as pivotal regulators of gene expression at the RNA level^5^. miRNAs suppress target gene expression by promoting mRNA degradation or inhibiting translation, thereby affecting a wide spectrum of biological processes such as development, differentiation, proliferation, and death^6^,^7^. It has been reported that miRNAs function as oncogenes or tumor suppressors, and aberrant expression of miRNAs is related to cancer progression via the regulation of cell growth, drug resistance, and metastasis^8^,^9^,^10^. Several reports have demonstrated that miRNAs including miR-431, miR-185-5p, miR-542-5p, and miR-339-5p are involved in the regulation of metastatic cancer cells^11^,^12^. Although several efforts have been made to control metastasis, the metastatic potential of cancer cells remains largely unknown.

p130Cas (breast cancer anti-estrogen receptor 1, BCAR1) is a member of the Crk-associated substrate (Cas) family and functions as an adaptor protein governing receptor-mediated signal transduction by regulating protein-protein interactions^13^,^14^. It has been reported that p130Cas promotes the growth and migration of cancer cells and its expression was found to be augmented in several cancers^14^,^15^,^16^,^17^. Since p130Cas has the potential as a proto-oncogene, the mechanisms regulating p130Cas expression and activity needs to be understood. Posttranslational regulation of p130Cas such as proteolytic cleavage or reversible phosphorylation of tyrosine residues are known to be essential for p130Cas activity^18^,^19^. In addition, miRNAs were also involved in the regulation of p130Cas expression; miR-362-3p and miR-329 suppressed cancer progression by targeting p130Cas^20^.

In this study, we investigated the role of miR-24-3p, one of the downregulated miRNAs in metastatic cancers, in the regulation of cell migration and invasion. Ectopic expression of miR-24-3p inhibited cell migration, growth, and drug sensitivity in five different cell lines including MCF7, Hep3B, B16F10, SH-Hep1, and PC-3 via p130Cas downregulation. miR-24-3p suppressed the translation of *p130Cas* mRNA and EGFP-Cas expression restored miR-24-3p-induced tumor suppressive effects. Taken together, our results suggest that miR-24-3p has a tumor suppressive role in cancer cells, and that the miR-24-3p/p130Cas axis regulates the metastatic potential of cancer cells.

## Materials and Methods

### Cell culture, transfection, plasmids and miRNAs

Human breast adenocarcinoma MCF7 cells, hepatocellular carcinoma Hep3B and SK-Hep1cells were cultured in Dulbecco’s modified essential medium (DMEM) (Hyclone, CA), supplemented with 10% fetal bovine serum and 1% penicillin/streptomycin at 37 °C in 5% CO_2_. Human prostate carcinoma PC-3 and mouse melanoma B16F10 cells were maintained in Roswell Park Memorial Institute medium (RPMI) (Hyclone, CA), supplemented with 10% fetal bovine serum and 1% penicillin/streptomycin. MCF7 clones stably expressing either pEGFP or pEGFP-p130Cas were also maintained in DMEM/10% FBS/1% penicillin/streptomycin with 0.5 mg/ml of G418^20^. EGFP reporter plasmids were cloned by inserting 3′UTR of human *p130Cas* mRNA (3002–3150 bp) into pEGFP-C1 (BD Bioscience, NJ) as described in a previous study^21^. A mutant reporter plasmid missing the miR-24-3p binding site was generated by site-directed mutagenesis using KOD plus mutagenesis kit (Toyobo, Japan). Plasmids and miRNAs (Bioneer, Korea) were transfected using Lipofectamin 2000 (Invitrogen, CA) according to the manufacturer’s instruction.

### RNA analysis

Total RNAs were isolated from cell lines using Trizol reagent (Invitrogen, CA). For the analysis of mRNA, complementary DNA (cDNA) was synthesized by reverse transcription using a ReverTra Ace^®^ RT Kit (Toyobo, Japan). For miRNA analysis, cDNA was prepared using the MiR-X™ miRNA First-Strand cDNA synthesis kit (Clonetech, CA) according to the manufacturer’s instructions. The relative abundance of each transcript was assessed by real-time quantitative polymerase chain reaction (RT-qPCR) using the Kapa SYBR Fast qPCR kit (Kapa Biosystems, MA) and specific primer sets on the StepOne Plus™ system (Applied Biosystems, CA). Primer sequences are listed in Table 1.

### Western blot analysis

Whole cell lysates were prepared using RIPA buffer containing 10 mM Tris–HCl (pH 7.4), 150 mM NaCl, 1% NP-40, 1 mM EDTA and 0.1% sodium dodecyl sulfate separated by electrophoresis in SDS-containing polyacrylamide gels (SDS-PAGE), and transferred onto PVDF membranes (Millipore, MA). The blots were incubated with the following antibodies against GFP (Santa Cruz biotechnology, TX), β-actin (Abcam, MA), p130Cas (Cas2)^22^, Vimentin (Santa Cruz biotechnology, TX), E-cadherin (BD Biosciences, NJ), and N-cadherin (Abcam, MA), then sequentially incubated with the appropriate secondary antibodies conjugated with horseradish peroxidase (HRP) (Santa Cruz Biotechnology, TX). Chemo-luminescent signals were visualized using NEW Clarity™ ECL substrate (Bio-Rad, CA).

### Nascent translation assay

*De novo* translation of p130Cas was estimated by incubating cells with 1 mCi L-[^35^S] methionine and L-[^35^S] cysteine (PerkinElmer Life Sciences, MA) for 20 min. After cell lysis, ^35^S-labeled p130Cas protein was immunoprecipitated using anti-Cas2 antibody, separated by SDS-PAGE, and transferred onto a PVDF membrane. Radioreactivity was visualized using the PharoseFX™ Plus System (Bio-Rad, CA)^21^.

### Scratch wound healing, migration and invasion assay

A wound healing assay was performed as previously described^23^. Confluent cancer cells were wounded using a pipette tip and cultured in DMEM/1% FBS media. Cell images were obtained with an IX71 inverted microscope (Olympus, Japan). For the migration and invasion assay, a transwell chamber was coated with or without matrigel solution and incubated for 90 min at 37 °C. After the transfection of miRNAs, cells were seeded in upper chambers with low-serum media and complete media was added to the lower chambers. Migrated cells were fixed and stained using the Diff-Quik Staining kit (Sysmex, Japan). Cell images were obtained with an Axiovert 200 inverted microscope (Zeiss, Germany).

### Cell growth assay

A colorimetric assay using the tetrazolium salt, 3-(4, 5-dimethylthiazol-2-yl)-2, 5-diphenyltetrazolium bromide (MTT) was used to assess cell viability. After miRNA transfection, cells were incubated with 0.5 mg/ml of MTT solution for 3 h at 37 °C. Formazan crystals were dissolved with isopropanol and the absorbance at 570 nm was measured using the VICTOR3 multi-label plate reader (Perkin-Elmer, MA). For the colony forming assay, 1 × 10^3^ cells were seeded in 6-well plates and cultured for 3 weeks. Then, the cells were fixed with 4% formaldehyde and stained with 0.05% crystal violet for 10 min at room temperature. Colonies from each well were counted from three random fields (100 mm^2^) per sample. Cell numbers were determined using the LUNA™ Automated Cell Counter (Logos Biosystems, VA) after trypan blue staining.

### Xenograft tumor growth assay

All animal experiments were performed according to approved protocols from IACUC at the College of Medicine, The Catholic University of Korea. After transfection of miRNAs, 2 × 10^6^ cells were mixed with matrigel (BD Biosciences, NJ) and implanted subcutaneously into the flank of BALB/c Nude mice (6 week old, male) (*n* = 5). After 4 weeks, the animals were sacrificed and the tumor masses were analyzed.

### *In vivo* metastasis assay

After transfection of B16F10 cells with miR-24-3p and control miRNA, cells (3 × 10^5^ cells/mouse) were injected into C57BL6 mice (*n* = 5) via the tail vein. Mice were sacrificed 17 days later and the lungs were fixed in 10% formaldehyde. The number of B16F10 colonies present on the surface of each set of lungs was determined by visual inspection.

### *In silico* analysis of differential expression of miRNAs

miRNA expression profiling data sets were obtained from the National Center for Biotechnology Information (NCBI) Gene Expression Omnibus (GEO) database portal (http://www.ncbi.nlm.nih.gov/geo/, Accession Number: GSE67139, GSE67138, GSE21036 and GSE31384). Relative expression of miR-24 in each data set was analyzed by comparing the values between metastatic and non-metastatic primary tumor specimens. The survival rates of patients were determined by the Kaplan-Meier estimate according to the relative levels of miR-24-3p.

## Results

### miR-24-3p was downregulated in metastatic cancer

**Figure 1:**
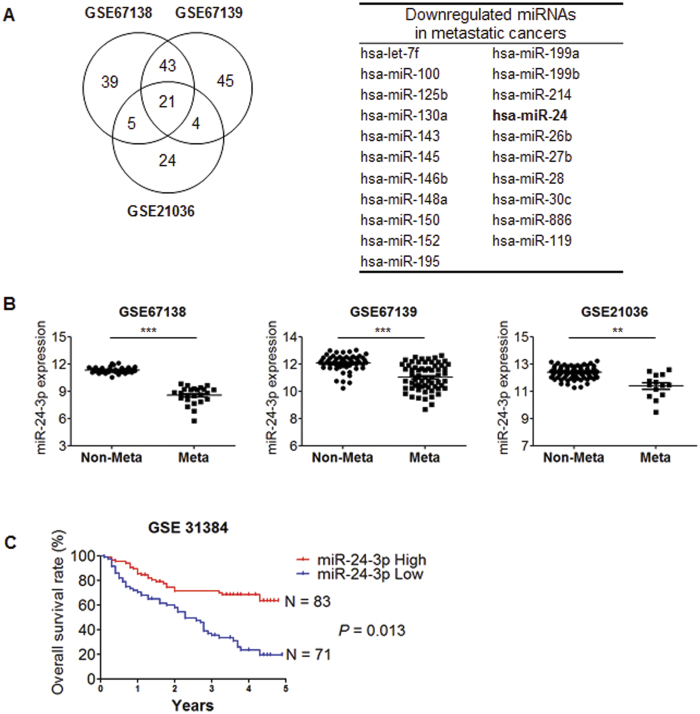
Differential expression of miR-24-3p in metastatic cancer. (**A**) Analysis of differentially expressed miRNAs in metastatic cancer using three different Gene Expression Omnibus databases (GES67138 and GES67139, human hepatocellular carcinoma; GES21306, human prostate cancer). The number indicates the downregulated miRNAs in metastatic cancers and the list of miRNAs is shown as a table. (**B**) The relative level of miR-24-3p in the metastatic group and the non-metastatic group was analyzed from three different sets of microRNA microarray data. (**C**) Kaplan-Meier survival curve. The relation between the overall survival rate of cancer patients and the miR-24-3p level was analyzed in the GES31384 dataset.

To identify metastasis-related miRNAs, we performed *in silico* cross analysis using three independent GEO data sets (GSE67138, GSE67139, and GSE21036) and analyzed the downregulated miRNAs in three different groups as listed in Fig. 1A. We further investigated the relative level of miR-24-3p between primary tumors without metastasis (Non-Meta) and metastatic tumors (Meta) and found that miR-24-3p was significantly downregulated in metastatic tumors (Fig. 1B). We also analyzed the overall survival rate of cancer patients depending on the miR-24-3p level and observed that the patients with lower expression of miR-24-3p showed poor survival (Fig. 1C). This survey indicates that decreased miR-24-3p is related to the metastasis of cancer cells.

### miR-24-3p inhibited cell migration and invasion of cancer cells

**Figure 2:**
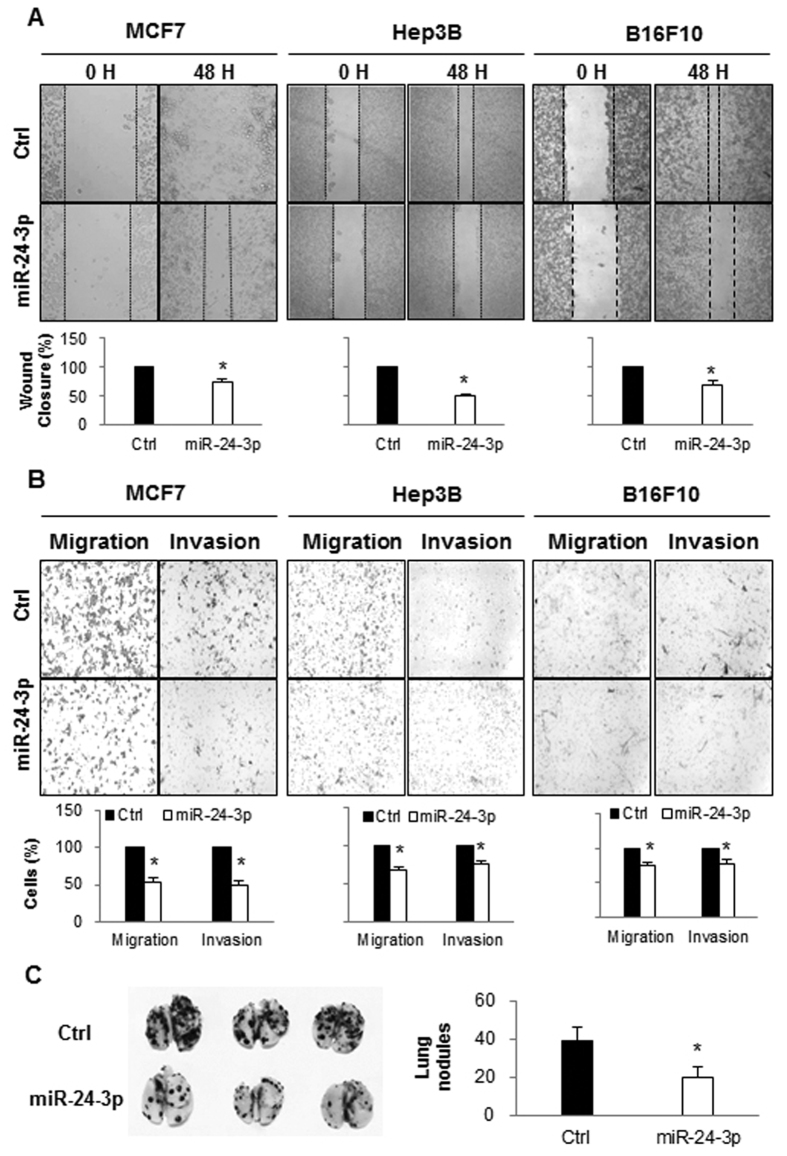
miR-24-3p suppressed migration and invasion of cancer cells. (**A**) Analysis of wound closure after miRNA transfection. MCF7, Hep3B, and B16F10 cells were transfected with either miR-24-3p mimic or control miRNAs and cultured until they reached confluency. After wounds were created, the cell migration distance was analyzed 48 h later. (**B**) Migration and invasion assay. After the transfection of miRNAs, cells were cultured in transwell with or without matrigel, and migrated cells were stained and analyzed by counting cells from three different fields. (**C**) After transfection of miR-24-3p and control miRNA, B16F10 cells (3 × 10^5^ cells/mouse) were injected into the tail vein of C57BL6 mice (*n* = 5). 17 days later, mice were sacrificed and the lungs were isolated. The number of B16F10 colonies present on the surface of each set of lungs was determined by visual inspection. Data are representative from three independent experiments. *p < 0.05.

Metastasis is a multistep process including cell migration, invasion of ECM membranes, and epithelial and mesenchymal transition (EMT)^24^,^25^,^26^. To test whether a lower level of miR-24-3p is related to an increase in the metastatic potential of cancer cells, we investigated cell migration and invasion in five different types of cancer cells, human breast adenocarcinoma MCF7 and human hepatocellular carcinoma Hep3B and SK-Hep1 cells, human prostate cancer PC-3 cells, and mouse melanoma B16F10 cells after ectopic expression of miR-24-3p. As shown in Fig. 2A and Supplementary Figure S1, miR-24-3p expression reduced the rate of wound closure in MCF7, Hep3B, B16F10, SK-Hep1, and PC-3 cells. Cell migration and invasion were further assessed using Transwell chambers with or without matrigel after the transfection of miR-24-3p. miR-24-3p overexpression resulted in a decrease in the cell migration and invasion ability of five different cancer cell lines (Figs 2B and S1). We further investigated whether miR-24-3p suppresses the ability of *in vivo* metastasis by tracing tail vein-injected B16F10 cells after miRNA transfection. The number of colonies on the surface of the lungs was smaller in miR-24-3p transfected group compared to the control group (Fig. 2C). We also investigated the levels of EMT maker proteins including vimentin, N-cadherin, and E-cadherin after miR-24 transfection. There were no significant changes in the levels of vimentin, N-cadherin, or E-cadherin (Supplementary Figure S2). These results suggest that miR-24-3p suppresses cell migration, invasion, and *in vivo* metastatic potential of cancer cells.

### miR-24-3p suppressed growth of cancer cells

**Figure 3:**
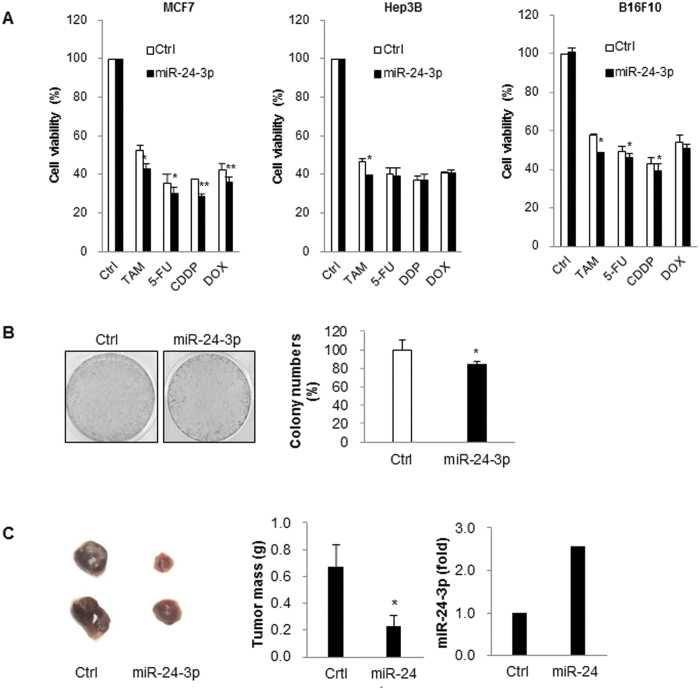
miR-24-3p suppressed growth of cancer cells. (**A**) After the transfection of miRNAs, MCF7, Hep3B, and B16F10 cells were cultured with or without the anti-cancer drugs including tamoxifen, 5-FU, CDDP, and doxorubicin, and cell viability was assessed by MTT assay. (**B**) MCF7 cells transfected with either miR-24-3p or control miRNA and 1 × 10^3^ cells were cultured for 3 weeks to form colonies. Colonies were fixed and stained with cystal violet solution and the number of colonies was counted. (**C**) miRNA-transfected MCF7 cells were transplanted into the mice (*n* = 5) and tumor development was observed over 4 weeks. Xenograft tumor mass and relative miRNA levels in tumors were analyzed from 4 different mice. Data are representative or indicate the mean ± SEM of three independent experiments. *p < 0.05; **p < 0.01.

To understand whether miR-24-3p regulates cell growth, we investigated cell viability and xenograft tumor growth after miR-24-3p transfection. We observed that miR-24-3p overexpression reduced cell viability in various types of cancer cells using the MTT assay (Figs 3A and S3). Also, miR-24-3p sensitized cancer cells in response to anti-cancer drugs including tamoxifen, 5-fluorouracil, CDDP, and doxorubicin (Figs 3A and S3). We also investigated the effect of miR-24-3p in the regulation of cell growth. After transfection of miR-24-3p and control miRNA, colony formation and xenograft tumor growth were assessed using MCF7 cells. miR-24-3p slightly decreased the number of colonies (Fig. 3B) and as well as the mass of xenograft tumors (*n* = 5) (Fig. 3C). Taken together, these observations suggest that miR-24-3p inhibits cell growth.

### miR-24-3p suppressed p130Cas expression

**Figure 4:**
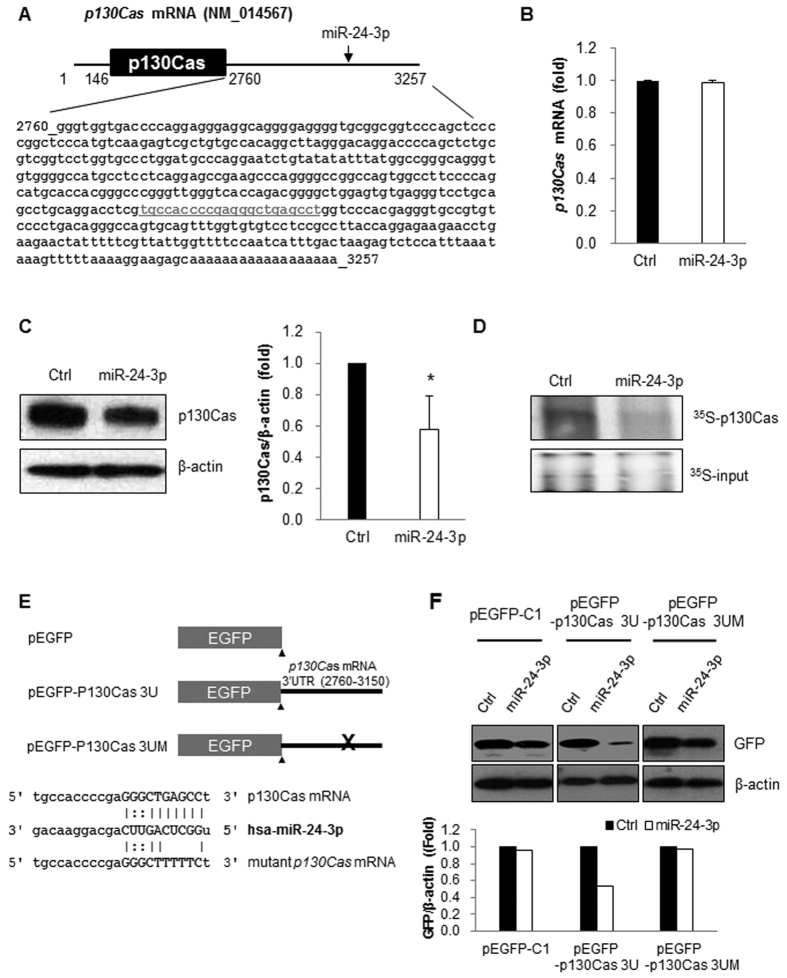
miR-24-3p down-regulated p130Cas expression. (**A**) Schematic diagram of *p130Cas* mRNA with miR-24-3p binding site. The predicted miR-24-3p binding site was found in the 3′UTR of *p130Cas* mRNA (underlined). (**B** and **C**) After the transfection of miRNAs, p130Cas mRNA and protein levels were determined by RT-qPCR (**B**) and Western blotting (**C**), respectively. GAPDH mRNA or β-actin was used as the internal control. (**D**) Translational regulation of p130Cas by miR-24-3p. After the incubation of miRNA-transfected MCF7 cells with ^35^S-methionine/cysteine, ^35^S-p130Cas protein was isolated using p130Cas antibody, separated by SDS-PAGE and visualized using PharoseFX Plus. The representative image shows relative levels of nascent p130Cas. (**E**) A schematic of the EGFP-reporter constructs. The 3′UTR of *p130Cas* mRNA containing a miR-24-3p binding site (3002–3150 nt) was inserted into pEGFP-C1 and a mutant reporter construct lacking the miR-binding site was generated using site-directed mutagenesis. (**F**) Reporter analysis after the miR-24-3p expression. After transfection of MCF7 cells with miRs and reporter constructs, EGFP levels were evaluated by Western blotting. β-actin was used as a loading control. All data are presented as mean ± SD of three independent experiments. *p < 0.05.

To determine the direct target of miR-24-3p, we performed *in silico* analysis using three different miRNA target prediction algorithms (miRwalk, Targetscan, and miRNA.org) and identified *p130Cas* mRNA as a novel target of miR-24-3p (Fig. 4A). Our previous studies and others indicated that p130Cas is a proto-oncogene governing cell migration/invasion and its differential expression is related to cancer progression^15^,^16^,^20^,^27^. To test whether miR-24-3p regulates p130Cas expression, we investigated p130Cas expression in MCF7 cells after the transfection of miR-24-3p. While the mRNA level of *p130Cas* was not affected by miR-24-3p overexpression (Fig. 4B), p130Cas protein was downregulated by miR-24-3p (Figs 4C and S4). Because we observed the downregulation of p130Cas protein by miR-24-3p without significant change of its mRNA level, we assumed that miR-24-3p mediates the translational repression of *p130Cas* mRNA and investigated the *de novo* synthesis of p130Cas after miR-24-3p overexpression by analyzing the incorporation of ^35^S-labeled Cys/Met into p130Cas during translation. As expected, miR-24-3p decreased the amount of newly synthesized ^35^S-p130Cas in MCF7 cells (Fig. 4D). These results indicate that miR-24-3p downregulates p130Cas via translational repression. To further confirm the regulation of p130Cas by miR-24-3p, we constructed an EGFP-reporter containing 149 bp of *p130Cas* mRNA 3′UTR (positions 3002-3150; pEGFP-p130Cas 3U) and a mutant EGFP reporter missing miRNA binding sites (pEGFP-p130Cas 3UM) (Fig. 4E). We analyzed the relative expression of the EGFP reporters after miR-24-3p overexpression and found that miR-24-3p decreased the level of the EGFP-p130Cas 3U but not that of the EGFP control or EGFP-p130Cas 3UM (Fig. 4F). Taken together, these results indicate that p130Cas is a novel target of miR-24-3p.

### miR-24-3p inhibited cell migration and invasion via p130Cas

**Figure 5:**
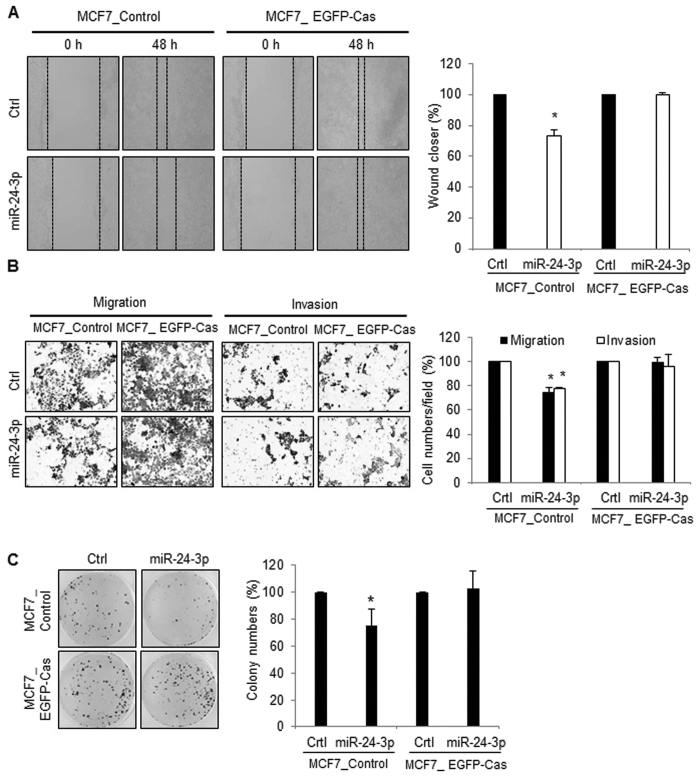
miR-24-3p suppressed cancer progression via p130Cas regulation. (**A** and **B**) MCF7_Control and MCF7_EGFP-Cas cells were transfected with either miR-24-3p mimic or control miRNAs and cell migration/invasion was analyzed. After the cells reached confluency, wound closure was determined by measuring cell migration distance (**A**). Cells were cultured in transwell with or without matrigel, and migrated cells were stained and analyzed by counting cells from three different fields (**B**). (**C**) 1 × 10^3^ of miRNA-transfected cells were cultured for 3 weeks. The colonies were stained with crystal violet and counted in 3 randomly selected visual fields. The images were representative of three independent experiments and graphs indicate the mean ± SEM of three independent experiments. *p < 0.05.

Since we observed that miR-24-3p has the potential to inhibit the cell migration/invasion and growth of cancer cells (Figs 2 and 3) and that p130Cas is a novel target of miR-24-3p (Fig. 4), we further investigated whether the negative regulation of miR-24-3p on cell migration/invasion was mediated by direct targeting of p130Cas. We generated stable MCF7 cell lines expressing EGFP or EGFP-Cas (MCF7_Control or MCF7_EGFP-Cas)^20^ and assessed the cell migration after miR-24-3p overexpression in stable cell lines. As shown in Fig. 5A, miR-24-3p overexpression resulted in a reduction of wound closure only in the MCF7_Control cells, but not in the MCF7_EGFP-Cas cells. In addition, inhibitory effects from miR-24-3p on cell migration, invasion, and colony formation were not observed in MCF7_EGFP-Cas cells (Fig. 5B and C). These results suggest that miR-24-3p suppresses cell migration and invasion via p130Cas regulation. Taken together, miR-24-3p negatively regulates cell migration/invasion by decreasing p130Cas level and the miR-24-3p/p130Cas axis has a novel role in the regulation of the metastatic potential of cancer cells.

## Discussion

miRNAs are involved in cancer progression by regulating a wide spectrum of cellular processes including growth, proliferation, metastasis, invasion, and drug sensitivity^9^,^10^,^28^. Aberrant expression of certain miRNAs is one of the hallmarks of cancer and can induce the downregulation of tumor suppressive miRNAs or an increase in oncogenic miRNAs that promote cancer development^11^,^12^,^20^. The migration/invasion ability of cancer cells is essential for cancer progression including metastasis leading to cancer-related death. Although several attempts have been made to overcome cancer metastasis, it still remains a major obstacle in cancer therapy. In this study, we investigated the roles of miR-24-3p as a downregulated miRNA in metastatic cancer. We observed that a lower level of miR-24-3p is related to the poor survival of cancer patients and that ectopic expression of miR-24-3p reduced the metastatic potential of cancer cells by suppressing cell migration, invasion, and proliferation. Furthermore, we identified p130Cas as a direct target of miR-24-3p and showed that the miR-24-3p/p130Cas axis is a novel factor governing cell migration and invasion.

miR-24-3p has been reported to function as an active regulator in a variety of cell types through different mechanisms. miR-24s (miR-24-1 and miR-24-2) are encoded in chromosomes 9 and 19 as a cluster with miR-23 and miR-27, respectively^29^. The sequences of miR-24-3p encoded in miR-24-1 and miR-24-2 genes are identical^30^,^31^. Several studies have shown that miR-24 can be a tumor suppressor as well as an oncogene. Specially, miR-24-3p suppressed cell proliferation and migration in prostate cancer^32^, arterial smooth muscle cells^33^, vascular muscle cells^34^, nasopharyngeal carcinoma^35^, bladder cancer^36^, and small-cell lung cancer^37^, but promoted the growth of glioma^38^, myeloid cells^39^, and squamous cell carcinoma^40^. The discrepancy in the role of miR-24-3p may be a result of the relative levels and differential regulation in different cell types. Regulatory mechanisms governing miR-24 expression in pathophysiological conditions are not fully elucidated. In this study, we investigated the relative level of miR-24-3p in human breast cancer tissues and corresponding non-cancer tissues; however, we did not observe differential expression of miR-24-3p in cancer cells (data not shown). Also, we analyzed the level of primary transcripts and the methylation state of the promoter region and found no significant changes between normal and cancer tissues (data not shown). Analysis of miR-24-3p expression between metastatic cancer and non-metastatic cancer tissues requires further investigation.

p130Cas functions as an integrator of cellular signaling that is essential for cell survival, migration, invasion, and proliferation by mediating protein-protein interactions^13^,^15^,^17^,^41^. Although the augmented expression of p130Cas in certain types of cancer is related to anti-cancer drug resistance and poor prognosis, the regulatory mechanisms are not fully elucidated. Reversible phosphorylation or proteolytic cleavage of p130Cas protein is known to be important for the fine-tuning of p130Cas-mediated signal transduction, which affects cell growth and migration^18^,^19^,^42^. Our recent report also showed that miR-362-3p and miR-329 are responsible for p130Cas regulation at the posttranscriptional level^20^. Here, we demonstrated that miR-24-3p is a novel regulator governing p130Cas expression. Since p130Cas plays an important role in cancer development, understanding the detailed mechanism of p130Cas regulation would provide a useful strategy for cancer therapy by targeting p130Cas^27^,^43^.

In this study, we hypothesized that miR-24-3p, a down-regulated miRNA in metastatic cancer, functions as a tumor suppressor by directly targeting p130Cas. Overexpression of miR-24-3p suppressed cell migration, invasion, and proliferation of cancer cells in a p130Cas-dependent manner. Therefore, the miR-24-3p/p130Cas axis is a novel regulator of cancer development and modulation of this axis may provide novel insights that may be helpful to regulate the metastatic potential of cancer cells.

## Additional Information

**How to cite this article:** Kang, H. *et al*. The miR-24-3p/p130Cas: a novel axis regulating the migration and invasion of cancer cells. *Sci. Rep.* **7**, 44847; doi: 10.1038/srep44847 (2017).

**Publisher's note:** Springer Nature remains neutral with regard to jurisdictional claims in published maps and institutional affiliations.

## Supplementary Material

Supplementary Information

## Figures and Tables

**Table 1:** Primer sequence for RT-qPCR.

Primer	Sequence
miR-24-3p Fwd	AACACACCTATTCAAGGATTCA
miR-24-3p Rev	mRQ 3′primer (Clontech)
p130Cas Fwd	ATGGGCAGTACGAGAACAGC
p130Cas Rev	GGCCAGGTCGTGGTCTATG
GAPDH Fwd	TGCACCACCAACTGCTTAGC
GAPDH Rev	GGCATGGACTGTGGTCATGAG
