# Author Correction: The miR-24-3p/p130Cas: a novel axis regulating the migration and invasion of cancer cells

**DOI:** 10.1038/s41598-021-90393-2

**Published:** 2021-05-26

**Authors:** Hoin Kang, Jun Gi Rho, Chongtae Kim, Hyosun Tak, Heejin Lee, Eunbyul Ji, Sojin Ahn, A-Ri Shin, Hyun-Il Cho, Yun Hyun Huh, Woo Keun Song, Wook Kim, Eun Kyung Lee

**Affiliations:** 1 Department of Biochemistry, College of Medicine, The Catholic University of Korea, Seoul, South Korea; 2 Department of Molecular Science and Technology, Ajou University, Suwon, South Korea; 3 Catholic Cancer Research Institute, College of Medicine, The Catholic University of Korea, Seoul, South Korea; 4 Department of Life Science, Bio Imaging and Cell Dynamics Research Center, Gwangju Institute of Science and Technology, Gwangju, South Korea; 5 Cancer Evolution Research Center, The Catholic University of Korea, Seoul, South Korea

Correction to: *Scientific Reports* 10.1038/srep44847, published online 24 March 2017

This article contains errors in MCF7 cell images of Figures 2 and 5. The correct Figures 2 and 5 appear below as Figures 1 and 2.

**Figure 1:**
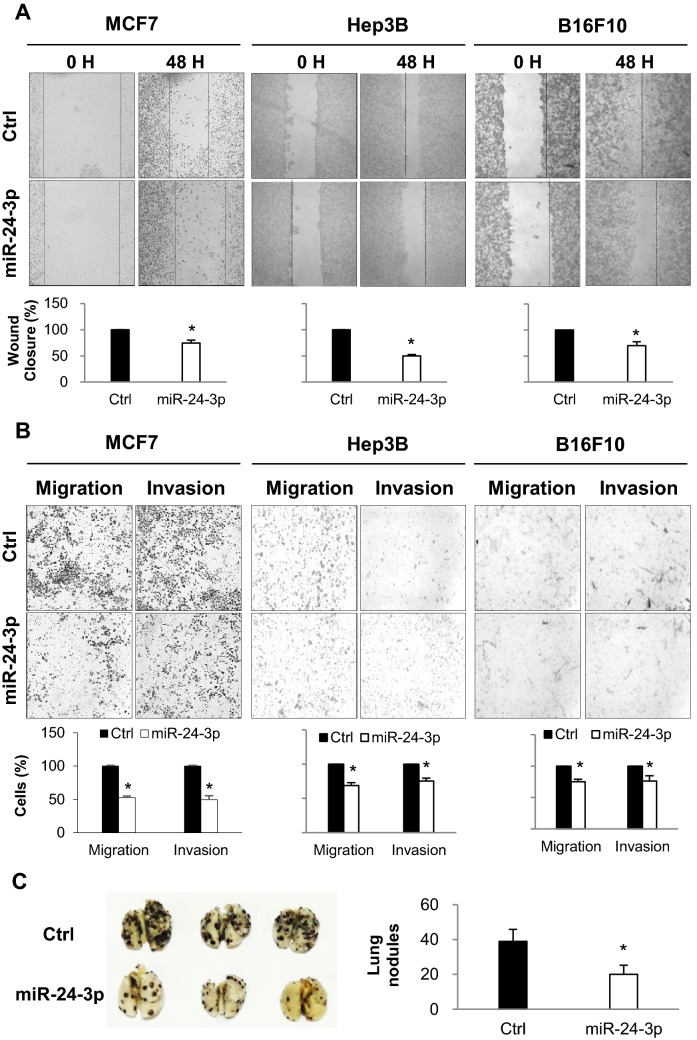
(**A**) Analysis of wound closure after miRNA transfection. MCF7, Hep3B, and B16F10 cells were transfected with either miR-24-3p mimic or control miRNAs and cultured until they reached confluency. After wounds were created, the cell migration distance was analyzed 48 h later. (**B**) Migration and invasion assay. After the transfection of miRNAs, cells were cultured in transwell with or without matrigel, and migrated cells were stained and analyzed by counting cells from three different fields. (**C**) After transfection of miR-24-3p and control miRNA, B16F10 cells (3 × 10^5^ cells/mouse) were injected into the tail vein of C57BL6 mice (*n* = 5). 17 days later, mice were sacrificed and the lungs were isolated. The number of B16F10 colonies present on the surface of each set of lungs was determined by visual inspection. Data are representative from three independent experiments. *p < 0.05.

**Figure 2:**
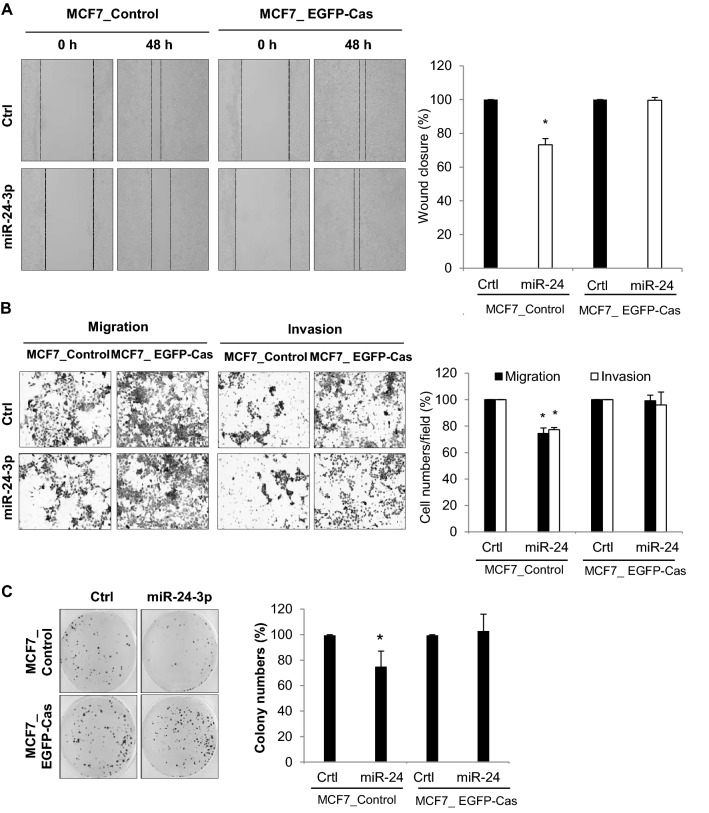
(**A**,**B**) MCF7_Control and MCF7_EGFP-Cas cells were transfected with either miR-24-3p mimic or control miRNAs and cell migration/invasion was analyzed. After the cells reached confluency, wound closure was determined by measuring cell migration distance (**A**). Cells were cultured in transwell with or without matrigel, and migrated cells were stained and analyzed by counting cells from three different fields (**B**). (**C**) 1 × 10^3^ of miRNA-transfected cells were cultured for 3 weeks. The colonies were stained with crystal violet and counted in 3 randomly selected visual fields. The images were representative of three independent experiments and graphs indicate the mean ± SEM of three independent experiments. *p < 0.05.

