# Supplementary material for: The miR-24-3p/p130Cas: a novel axis regulating the migration and invasion of cancer cells
Source: Sci Rep. 2017 Mar 24;7:44847. doi: 10.1038/srep44847 (PMC5364481; doi:10.1038/srep44847)

The miR-24-3p/p130Cas: a novel axis regulating the migration and invasion of cancer cells

Hoin Kang[1], Jun Gi Rho[2], Chongtae Kim[1], Hyosun Tak[1], Heejin Lee[1], Eunbyul Ji[1], Sojin Ahn[1], A-Ri Shin[3], Hyun-Il Cho[3], Yun Hyun Huh[4], Woo Keun Song[4], Wook Kim[2] *, Eun Kyung Lee[1,5]*

Supplementary Figure S1.

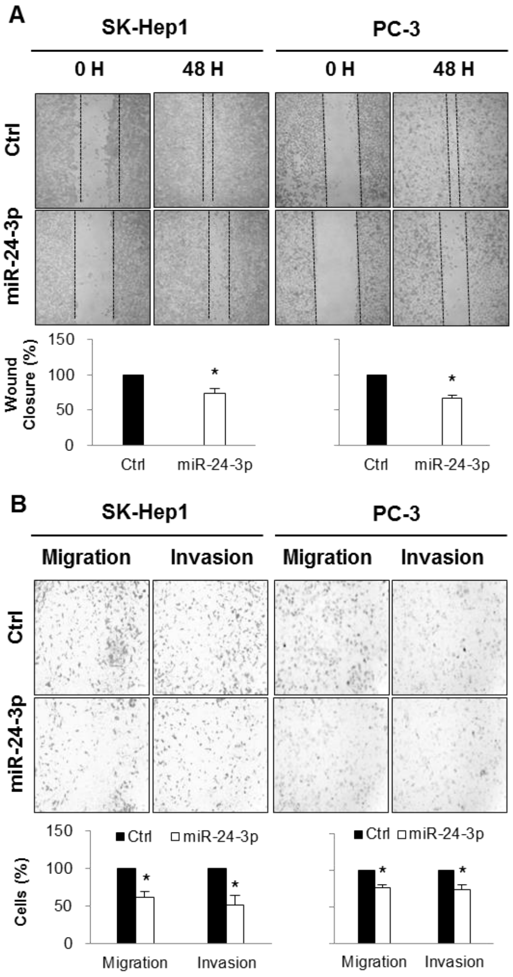

A) Analysis of wound closure after miRNA transfection. SK-Hep1 and PC-3 cells were transfected with either miR-24-3p mimic or control miRNAs and cultured until they reached confluency. After wounds were created, the cell migration distance was analyzed 48 h later. (B) Migration and invasion assay. After the transfection of miRNAs, cells were cultured in transwell with or without matrigel, and migrated cells were stained and analyzed by counting cells from three different fields. Images are representative from three independent experiments and graphs indicate the mean ± SEM of three independent experiments. *, $p<0.05$

Supplementary Figure S2.

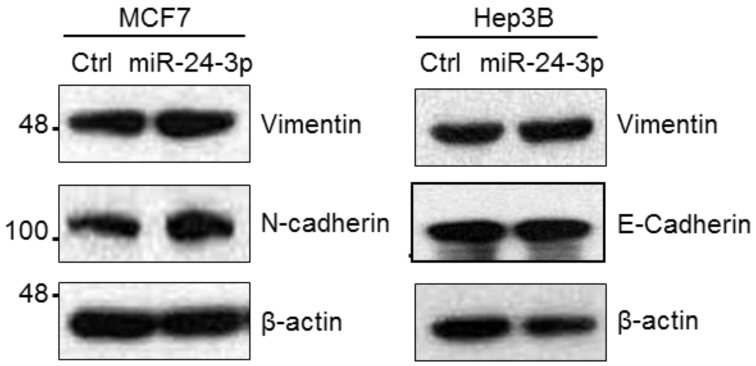

After the transfection of miRNAs, the expression of vimentin, N-cadherin and E-cadherin was assessed by Western blotting. β-actin was used as an internal control. The images were representative of three independent experiments.

Supplementary Figure S3.

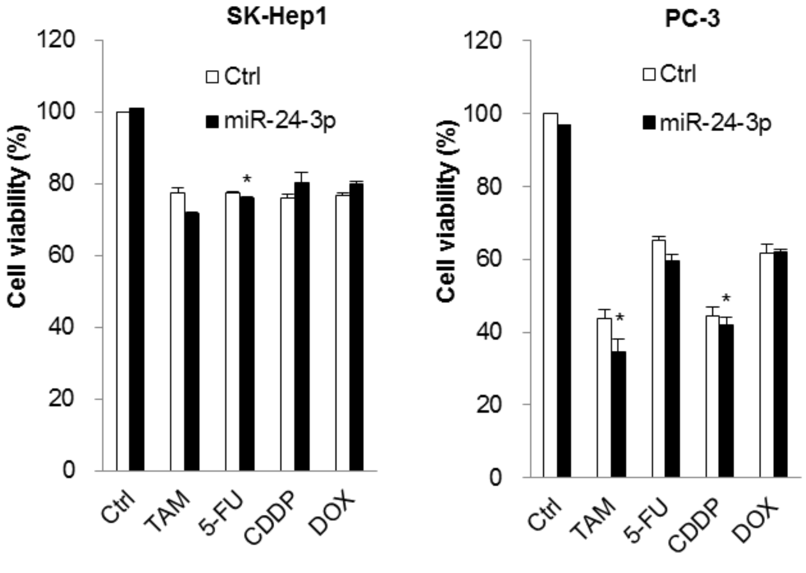

After transfection of miR-24-3p and control miRNA, SK-Hep1 and PC-3 cells were cultured with anti-cancer drugs including tamoxifen, 5-FU, CDDP, and doxorubicin. After 48 h, cell viability was assessed by MTT assay. Graphs indicate the mean ± SEM of three independent experiments. *, $p<0.05$

Supplementary Figure S4.

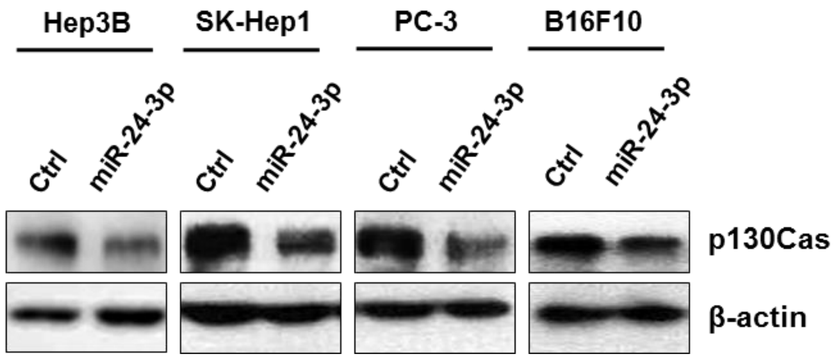

After transfection of miR-24-3p and control miRNAs into four different cancer cells, p130Cas levels were analyzed by western blotting. β-actin was used as a loading control.

Supplementary Figure S5.

Figure 4C

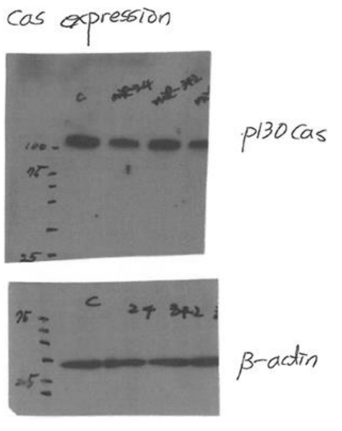

Figure 4F

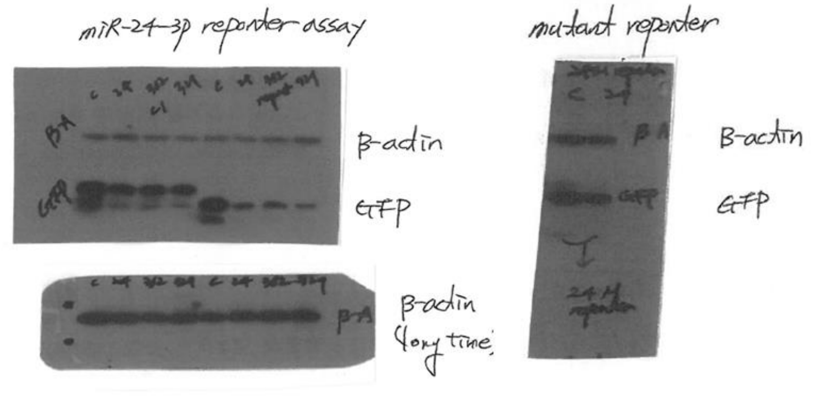

Supplementary figure 2

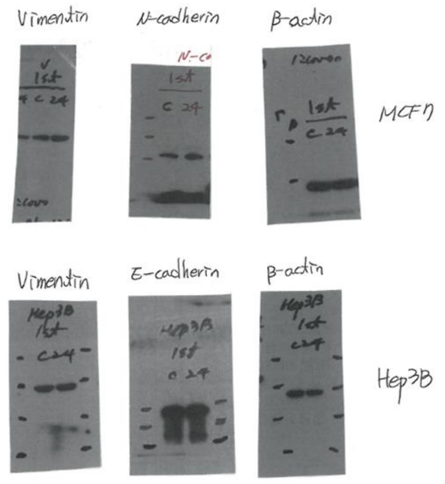

Supplementary figure 4

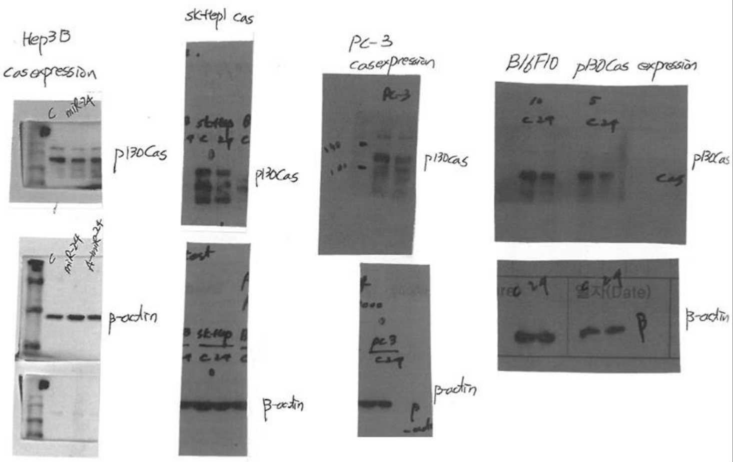

Supplement: Supplementary Information [file srep44847-s1.pdf]
